# Rational oral corticosteroid use in adult severe asthma: A narrative review

**DOI:** 10.1111/resp.13730

**Published:** 2019-11-12

**Authors:** Li Ping Chung, John W. Upham, Philip G. Bardin, Mark Hew

**Affiliations:** ^1^ Department of Respiratory Medicine Fiona Stanley Hospital Perth WA Australia; ^2^ Department of Respiratory Medicine Princess Alexandra Hospital and University of Queensland Brisbane QLD Australia; ^3^ Department of Respiratory and Sleep Medicine Monash Medical Centre, Monash University Melbourne VIC Australia; ^4^ Allergy, Asthma and Clinical Immunology Alfred Hospital Melbourne VIC Australia

**Keywords:** asthma, biological products, glucocorticoids, health promotion, morbidity

## Abstract

OCS play an important role in the management of asthma. However, steroid‐related AE are common and represent a leading cause of morbidity. Limited published studies suggest OCS usage varies across countries and recent registry data indicate that at least 25–60% of patients with severe asthma in developed countries may at some stage be prescribed OCS. Recent evidence indicate that many patients do not receive optimal therapy for asthma and are often prescribed maintenance OCS or repeated steroid bursts to treat exacerbations. Given the recent progress in adult severe asthma and new treatment options, judicious appraisal of steroid use is merited. A number of strategies and add‐on therapies are now available to treat severe asthma. These include increasing specialist referral for multidisciplinary assessments and implementing OCS‐sparing interventions, such as improving guideline adherence and add‐on tiotropium and macrolides. Biologics have recently become available for severe asthma; these agents reduce asthma exacerbations and lower OCS exposure. Further research, collaboration and consensus are necessary to develop a structured stewardship approach including realistic OCS‐weaning programmes for patients with severe asthma on regular OCS; education and public health campaigns to improve timely access to specialized severe asthma services for treatment optimization; and implementing targeted strategies to identify patients who warrant OCS use using objective biomarker‐based strategies.

## INTRODUCTION

Oral corticosteroids (OCS) have been the preferred treatment for acute asthma exacerbations since the 1950s.^1^ Systematic reviews have demonstrated the effectiveness of OCS for treating asthma exacerbations, reducing relapses, lowering short‐acting beta_2_‐agonist (SABA) use^2^ and reducing hospital admissions by 60% in the acute setting.^3^ Additionally, daily maintenance (long‐term, low‐dose) OCS is a guideline‐supported therapy for uncontrolled severe asthma, defined as poor control despite optimal therapy with high‐dose inhaled corticosteroid (ICS) and long‐acting β_2_‐agonist (LABA).^4^, ^5^, ^6^

Unfortunately, maintenance OCS use is a leading cause of serious drug‐related adverse effects (AE),^7^ and substantially increasing healthcare costs.^8^ Emerging evidence suggests that repetitive bursts of OCS for acute asthma exacerbations also lead to cumulative AE.^9^, ^10^ This is disturbing considering that a high proportion of severe asthma patients require ≥2 OCS bursts per year for managing exacerbations.^11^, ^12^, ^13^

Prior to the availability of new treatments, >50% of patients with uncontrolled severe asthma required maintenance OCS, in addition to conventional therapy to manage their symptoms.^11^ Multidimensional assessment (MDA)^14^, ^15^, ^16^, ^17^ and a number of new add‐on agents can reduce the frequency of asthma exacerbations, thereby reducing the need for OCS.^18^ Selected biologics also decrease maintenance OCS use in severe asthma.^19^, ^20^ However, ongoing high OCS use in severe asthma suggests these measures have not been optimally applied.^21^, ^22^, ^23^, ^24^

In rheumatology, the advent of novel treatments, together with improved care through multidisciplinary teams, has revolutionalized treatment and substantially reduced OCS use.^25^ Recognition of the equivalent problem in respiratory medicine coupled to appropriate remedial strategies can therefore help to optimize OCS use.^26^

The aim of the current review was first to examine the prevalence and morbidity associated with OCS for adult severe asthma. Second, we review current strategies effective in minimizing inappropriate OCS exposure. Finally, we highlight implementation barriers and propose strategies to limit health risks associated with OCS use in severe asthma.

## USAGE, PREVALENCE AND BURDEN OF OCS IN SEVERE ASTHMA

Frequent OCS bursts or maintenance OCS use is associated with numerous AE.^8^, ^27^, ^28^, ^29^ These AE substantially reduce the health‐related quality of life (HRQoL) of people with asthma,^22^, ^30^ and have major economic and societal consequences.^8^, ^27^, ^28^

### Usage and prevalence

Clinical guidelines, such as the Global Initiative for Asthma (GINA) guidelines^6^ and the Australian Asthma Handbook,^31^ recommend the addition of low‐dose maintenance OCS after all other conventional treatments have failed to control asthma, when adequate inhaler technique and adherence has been achieved, and after exclusion of other contributory factors. Additionally, short bursts of OCS (i.e. 5–10 days) are recommended to manage severe asthma exacerbations.^6^, ^31^

Unfortunately, studies reporting acute or chronic OCS usage in severe asthma are extremely limited and mostly retrospective. The literature for ‘chronic OCS use’ needs careful interpretation as some studies define this as daily maintenance therapy while others report chronic use based on repeated, cumulative OCS burst averaged over a year. In some instances, a combination of both criteria are used. For the purpose of this manuscript, the term chronic OCS use refers to maintenance OCS therapy; studies examining cumulative OCS exposure over time will be discussed separately.

On the basis of most recent Australian data, maintenance OCS are prescribed in up to 25% of severe asthma.^13^ The median daily dose for severe asthma is estimated to be 10 mg/day (prednisolone equivalent), although a wide dose range is reported (2–50 mg).^13^ Similar data have been reported from a difficult asthma registry, in which the median prednisolone dose was 10–15 mg/day across several specialist UK centres.^11^ A similar prevalence of maintenance OCS use in severe asthma was reported in a meta‐analysis of 26 studies examining the use and effect of MDA.^17^

In other countries, the use of OCS varies. The International Severe Asthma Registry reports that 59.6% of severe asthma patients in the UK were prescribed OCS maintenance treatment, while fewer patients used OCS in the USA (23.3%), South Korea (20.7%) and Italy (5.2%) (unpublished data presented at 2018 Respiratory Effectiveness Group Summit). Some retrospective studies report higher but variable OCS usage rates across different regions of the world (Table S1 in Supplementary Information).^23^

A longitudinal UK study from 2005 to 2012, involving >60 000 patients with severe asthma, found 75% of severe asthma patients were exposed to OCS.^32^ During 8 years of follow‐up, the proportion of patients exposed to an average cumulative OCS dose of up to 2.5 mg/day prednisolone equivalent increased with each year of study observation. In a US retrospective analysis of data from 2009 to 2010 (*n* = 9546), approximately, 40% of patients with persistent asthma of varying severity were prescribed OCS. Approximately, 8% were considered chronic OCS users, defined as either ≥2.5 mg/day of prednisolone equivalent or ≥4 OCS bursts/year.^33^

### Burden of chronic OCS

Chronic OCS use leads to various AE^27^ and recent evidence suggests that OCS is associated with a substantial excess mortality risk.^34^ A cross‐sectional study found 93% of patients with severe asthma had ≥1 condition linked to systemic corticosteroid (SCS) exposure, and 53% had ≥3 morbidities. The most prevalent comorbidities identified in this study are summarized in Table 1.^7^

**Table 1:** Prevalence of OCS‐related comorbidities in patients with severe asthma

OCS‐related comorbidities	Prevalence (%)
Dyspeptic disorders	65
Obesity (body mass index >30)	42
Psychiatric disorders	38
Hypertension	34
Osteoporosis	16
Hypercholesterolaemia	15
Type 2 diabetes	10
Osteopenia	10
Cardiovascular disease	10
Cataract	9
Fracture	5
Glaucoma	4
Sleep disorder	4

Prevalence data from the cross‐sectional Optimum Patient Care Research Database and the British Thoracic Society Difficult Asthma registry (*n* = 808) (Adapted from Sweeney *et al*.^7^).

The risk of OCS‐related AE, however, is non‐uniform across severe asthma populations, with younger patients (age ≤60 years) at greater odds for developing a broader range of AE.^35^ The greater burden of AE in younger patients correlates with escalating healthcare costs per patient per year.

A number of observational studies suggest a direct dose–response relationship between maintenance OCS use and complications, accompanied by a significant increase in asthma burden and healthcare costs.^8^, ^9^, ^27^, ^29^, ^32^, ^33^ In a retrospective analysis of 12 697 asthma patients treated with SCS (oral or parenteral) for ≥6 months and 12 697 non‐steroid users, patients with low SCS exposure (i.e. <5 mg prednisolone equivalent/day) had a 1.48‐fold risk of developing chronic AE (e.g. musculoskeletal, metabolic and psychiatric) relative to patients without exposure to SCS.^8^ In contrast, medium SCS exposure (i.e. prednisolone equivalent of ≥5–10 mg/day) and high SCS exposure (>10 mg/day) increased the risk of chronic AE by 2.19‐fold and 2.34‐fold, respectively.^8^

The cost of managing patients with severe refractory asthma on maintenance OCS is >40% higher than those not on maintenance corticosteroid. The additional costs were not only related to asthma medications, but also their non‐asthma medications required to manage OCS‐related AE, such as gastro‐oesophageal reflux and osteoporosis.^36^

### Cumulative burden of OCS bursts

Recent evidence suggests that even short burst of OCS can be associated with AE,^37^ and each OCS prescription results in a cumulative burden, regardless of the dose and duration.^10^ Longitudinal, retrospective data indicate AE increase in a dose‐dependent manner above cumulative exposures of 1.0–2.5 g prednisone equivalent, and for some outcomes at cumulative exposures of <1 g.^9^

A retrospective cohort study investigated the association between the number of OCS prescriptions and the incidence of AE.^10^ A total of 72 063 adults with variable asthma severity receiving an OCS prescription during the study period were matched with 156 373 non‐OCS users. Subjects taking ≥4 OCS prescriptions for maintenance or acute use had ≥1.29 times the odds of experiencing an AE in the current year compared to non‐OCS patients. The odds for a new AE was also marginally increased for those receiving one to three prescriptions within a year. These results suggest with increasing OCS prescription there is a cumulative risk of deleterious AE, regardless of whether OCS use is continuous or intermittent.

A population‐based cohort analysis compared the incidence of three acute AE (i.e. sepsis, venous thrombosis and fractures) in OCS users with non‐OCS users.^37^ The median prednisone equivalent daily dose was 20 mg/day and the median treatment duration was 6 days. The majority of patients (70%) received only one OCS course. Remarkably, despite the relatively brief treatment duration, there was a statistically significant increase in the rate of sepsis (incidence rate ratio (IRR): 3.77), venous thromboembolism (IRR: 3.11) and fracture (IRR: 1.96) in those who received OCS for respiratory conditions (e.g. asthma, upper or lower respiratory tract infections) versus non‐users within 30 days of steroid initiation. This is concerning considering the risk of respiratory infections or exacerbations needing OCS burst in severe asthma patients.

### Impact on quality of life

OCS‐related AE and morbidity have a major impact on the HRQoL of patients.^30^ Qualitative studies report a significant reduction in patient well‐being, often resulting in a greater burden than asthma itself.^30^ Fear of OCS morbidities is common among patients.^24^ Psychological AE, such as depression and irritability, and other common steroid complaints, such as weight gain, are distressing to patients, markedly impairing daily functioning^30^ and willingness to adhere to treatment,^22^ therefore worsening asthma symptoms. However, it may be difficult to distinguish the impact of OCS on HRQoL from that of severe asthma itself.

## EFFECTIVE OCS‐SPARING STRATEGIES

International guidelines recommend early specialist referral for patients with severe asthma to ensure accurate diagnosis and optimal management to improve asthma control and prevent exacerbations. However, some patients suffer frequent asthma exacerbations and are treated with multiple OCS bursts for several years before receiving specialist care,^38^, ^39^, ^40^, ^41^ increasing the risk of steroid‐related AE.

Asthma misdiagnosis and poor treatment adherence are relatively common, and may subject patients to unnecessary treatments including OCS.^42^, ^43^ In particular, suboptimal ICS use contributes to poor asthma control, and increases the risk of exacerbations necessitating OCS use or hospitalization.^6^, ^39^, ^44^ Effective strategies to address these challenges and reduce OCS exposure are discussed below.

### Multidimensional asthma assessment

**Figure 1:**
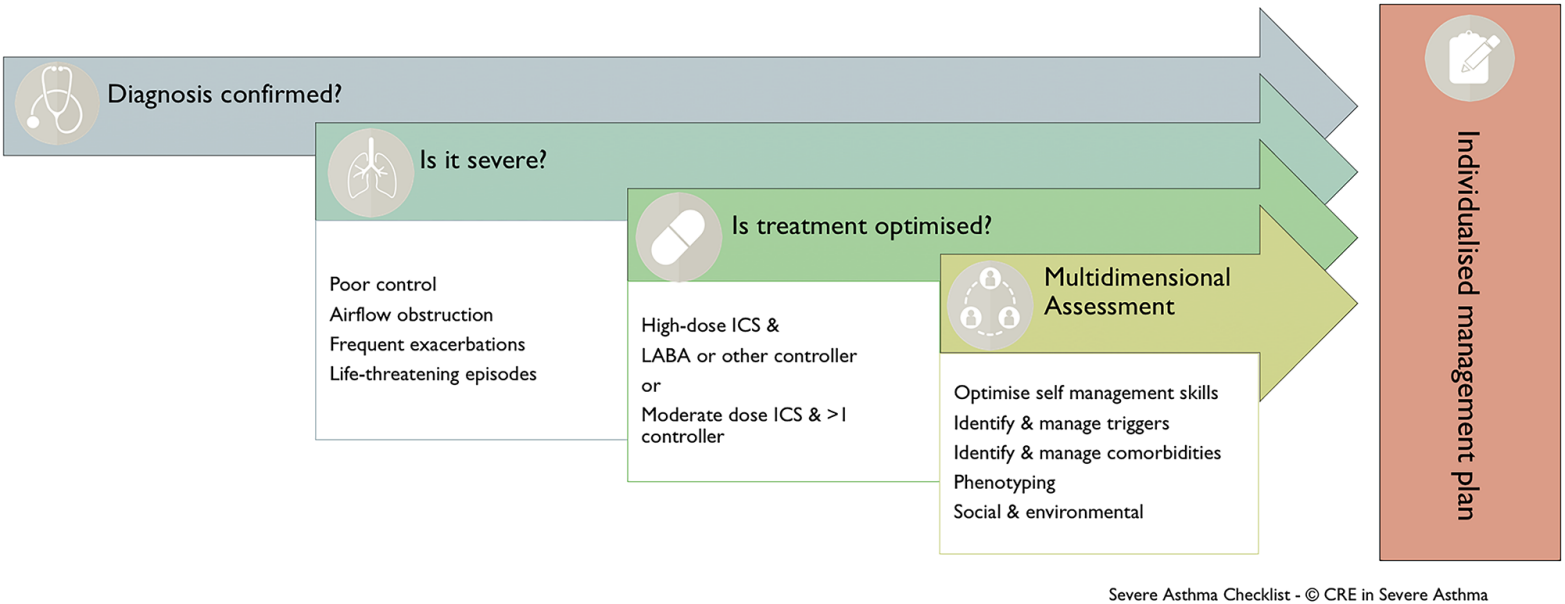
Multidimensional assessment approach to severe asthma (Reproduced with permission from the Centre of Excellence in Severe Asthma, originally developed as part of the Severe Asthma Toolkit (https://toolkit.severeasthma.org.au)). ICS, inhaled corticosteroid; LABA, long‐acting beta_2_‐agonist.

A multifaceted approach to systematically diagnose and characterize severe asthma,^14^ identify and treat risk factors and comorbidities^45^, ^46^ and target patients most likely to benefit from advanced therapies, such as biologicals, is crucial to reduce the overall steroid load (Fig. 1).

MDA improves asthma control and HRQoL, reduces OCS bursts by lowering asthma exacerbations and decreases hospitalizations.^14^, ^15^, ^16^, ^17^ Among patients evaluated through 10 UK Difficult Asthma Services, there was a lower corticosteroid burden at follow‐up compared to baseline.^16^ Fewer subjects required short‐burst therapy (77.4% vs 90.8%; *P* = 0.01) and the number of steroid courses was halved. Although the proportion of patients requiring maintenance OCS was unchanged, there was a reduction in steroid dose at follow‐up compared with baseline (10 vs 15 mg; *P* = 0.003).

### Biologicals

Commercially available humanized monoclonal antibodies target key cytokines of the allergic and eosinophilic pathways in asthma pathogenesis including immunoglobulin E (IgE), interleukin (IL)‐5 (IL‐5) and IL‐4/IL‐13.

#### *Reduction in asthma exacerbations*

Randomized, controlled trials with omalizumab, mepolizumab, benralizumab, reslizumab and dupilumab show a reduction in exacerbation rates, thereby reducing the need for OCS bursts and lowering total OCS cumulative exposure (Table 2).^47^, ^48^, ^49^, ^50^, ^51^, ^52^, ^53^

**Table 2:** Reduction in exacerbations with biologicals from randomized, placebo‐controlled, registration trials in severe asthma

Study name	Patient population	Intervention dose/duration	Reduction in clinically significant asthma exacerbations (primary outcome)			
			Intervention arm	Exacerbation rate†	Reduction vs placebo (%)	*P*‐value
INNOVATE^49^	12–75 years with severe allergic asthma	Omalizumab SC every 2–4 weeks administered for 28 weeks‡	Placebo (*n* = 210)	0.91	—	—
			Omalizumab (*n* = 209)	0.68	26	0.042
DREAM^48^	12–74 years with severe eosinophilic asthma	Mepo 75 mg IV; Mepo 250 mg IV; Mepo 750 mg IV for 13 months§	Placebo (*n* = 155)	2.4	—	—
			Mepo 75 mg (*n* = 153)	1.24	48	<0.0001
			Mepo 250 mg (*n* = 152)	1.46	39	0.0005
			Mepo 750 mg (*n* = 156)	1.15	52	<0.0001
MENSA^47^	12–82 years with severe eosinophilic asthma	Mepo 75 mg IV Q4W; Mepo 100 mg SC Q4W for 32 weeks§	Placebo (*n* = 191)	1.74	—	—
			Mepo IV (*n* = 191)	0.93	47	<0.001
			Mepo SC (*n* = 194)	0.83	53	<0.001
CALIMA^50^	12–75 years with severe eosinophilic asthma	Benra 30 mg SC Q4W; Benra 30 mg SC Q8W for 56 weeks¶	Placebo (*n* = 248)	0.93	—	—
			Benra 30 mg Q4W (*n* = 241)	0.60	33	0.0018
			Benra 30 mg Q8W (*n* = 239)	0.66	26	0.0188
SIRROCO^51^	12–75 years with severe eosinophilic asthma	Benra 30 mg SC Q4W; Benra 30 mg SC Q8W for 48 weeks¶	Placebo (*n* = 267)	1.33	—	—
			Benra 30 mg Q4W (*n* = 275)	0.73	45	<0.0001
			Benra 30 mg Q8W (*n* = 267)	0.65	51	<0.0001
Castro *et al*.^52^ study 1	12–75 years with severe eosinophilic asthma	Reslizumab 3 mg/kg IV Q4W or 52 weeks	Placebo (*n* = 132)	1.8	—	—
			Reslizumab (*n* = 92)	0.9	50	<0.0001
Castro *et al*.^52^ study 2	12–75 years with severe eosinophilic asthma	Reslizumab 3 mg/kg IV Q4W for 52 weeks	Placebo (*n* = 105)	2.11	—	—
			Reslizumab (*n* = 59)	0.86	59	<0.0001
LIBERTY ASTHMA QUEST^53^	≥12 years with uncontrolled moderate‐to‐ severe asthma	Dupi 200 mg (loading dose, 400 mg) or 300 mg (loading dose, 600 mg) SC Q2W for 52 weeks††	Placebo (*n* = 317)	0.87	—	—
			Dupi 200 mg Q2W (*n* = 631)	0.46	48	<0.001
			Placebo (*n* = 321)	0.97	—	—
			Dupi 300 mg Q2W (*n* = 633)	0.52	46	<0.001

† Annualized exacerbation rate, except for INNOVATE, which had exacerbation rate during the 28‐week treatment phase.

‡ Omalizumab dose based on patient's body weight and total serum IgE level at screening (≥0.016 mg/kg per IU/mL of IgE).

§ The approved dose of Mepo is 100 mg SC once Q4W.

¶ The approved dose of Benra is 30 mg SC once Q4W for the first three doses and then once Q8W thereafter. The first three doses of Benra were administered once Q4W.

†† In the USA, the approved dose of Dupi is an initial dose of 400 mg (two 200 mg injections) followed by 200 mg given every other week or an initial dose of 600 mg (two 300 mg injections) followed by 300 mg given every other week.

Benra, benralizumab; Dupi, dupilumab; Ig, immunoglobulin; i.v., intravenous; Mepo, mepolizumab; Q2W, every 2 weeks; Q4W, every 4 weeks; Q8W, every 8 weeks; SC, subcutaneous.

#### *Reduction in maintenance OCS*

Randomized, placebo‐controlled trials demonstrate mepolizumab, benralizumab and dupilumab also reduce maintenance OCS dosing in severe asthma (Table 3). The median reduction in OCS dose from baseline with mepolizumab was 50%. Complete steroid withdrawal was achievable in 14% versus 8% of patients.^19^ In patients treated with benralizumab, the median reduction in OCS dose was 75%, and >50% were withdrawn from OCS.^20^ In dupilumab‐treated patients, the OCS dose was reduced by 70%, and 48% discontinued OCS.^54^ The respective percentage reduction for placebo varied considerably across studies but often tended to be higher than anticipated (Table 3).

**Table 3:** Reduction in maintenance OCS dosing with biologicals from randomized, placebo‐controlled registration trials in severe asthma

Study name	Intervention dose/duration	Intervention	Reduction in daily OCS dose from baseline (%)†	*P‐*value	Patients achieving reduction in daily OCS dose from baseline by percentage category (%)			
					≥50% Reduction	≥75% Reduction	≥90% Reduction	100% Reduction
SIRIUS^19^	Mepolizumab 100 mg SC Q4W for 20 weeks‡	Placebo (*n* = 66)	0	—	33	18	11	8
		Mepolizumab (*n* = 69)	50	0.007	54	41	23	14
ZONDA^20^	Benralizumab 30 mg SC Q4W or Q8W for 28 weeks§	Placebo (*n* = 75)	25	—	35	20	12	19
		Benralizumab Q4W (*n* = 72)	75	<0.001	67	53	33	56¶
		Benralizumab Q8W (*n* = 73)	75	<0.001	66	52	37	52¶
LIBERTY ASTHMA VENTURE^54^	Dupilumab 300 mg SC Q2W (after a 600‐mg loading dose) for 24 weeks††	Placebo (*n* = 107)	42	—	53	39	31	29
		Dupilumab (*n* = 103)	70	<0.001	80	69	55	52

† Median dose reduction (SIRIUS and ZONDA); least‐squares mean dose reduction (LIBERTY ASTHMA VENTURE).

‡ The approved dose of mepolizumab is 100 mg SC once Q4W.

§ The approved dose of benralizumab is 30 mg SC once Q4W for the first three doses and then once Q8W thereafter.

¶ Patients with a baseline OCS dose of ≤12.5 mg per day at the end of the run‐in phase were eligible for a 100% dose reduction (discontinuation of oral glucocorticoid therapy).

†† In the USA, the approved dose of dupilumab is an initial dose of 400 mg (two 200 mg injections) followed by 200 mg given every other week or an initial dose of 600 mg (two 300 mg injections) followed by 300 mg given every other week.

OCS, oral corticosteroid; Q2W, every 2 weeks; Q4W, every 4 weeks; Q8W, every 8 weeks; SC, subcutaneous.

Evidence for omalizumab reduction maintenance OCS use is less compelling. A recent retrospective analysis found a lower likelihood of new OCS prescriptions with omalizumab treatment (OR: 0.55; 95% CI: 0.41–0.82).^55^ However, a 2014 Cochrane analysis reported no benefit on either the median daily dose or the number of participants who were able to withdraw from OCS treatment.^56^

Despite the proven OCS‐sparing effect with selected biologics, up to 25% of patients remain OCS‐dependent, highlighting the need for alternative strategies.^13^

### Other therapeutic options to reduce asthma exacerbations

Several other therapies can optimize asthma control and reduce acute exacerbations, potentially limiting OCS bursts. However, definitive data that macrolides or tiotropium have an OCS‐sparing effect require further clinical trials.

#### *Medium‐high dose ICS/LABA or ICS/formoterol maintenance and reliever*

Medium‐ or high‐dose ICS/LABA and ICS/formoterol as single maintenance and reliever therapy are the recommended GINA Step 4 treatments for patients with severe asthma. The latter approach prolongs time to first exacerbation and reduces the overall risk of exacerbation requiring OCS, emergency presentation or hospitalization by ≥25% compared with fixed ICS/LABA dosing.^57^

#### *Macrolides*

A randomized, double‐blind, placebo‐controlled trial showed that add‐on azithromycin 500 mg three times weekly in adults with persistent symptomatic asthma, despite ICS and LABA maintenance therapy, reduced the frequency of asthma exacerbations by 41% over 48 weeks versus placebo (*P* < 0·0001).^58^

#### *Tiotropium*

Add‐on tiotropium in two replicate, randomized, placebo‐controlled trials involving 912 patients with uncontrolled asthma despite high‐dose ICS and LABA increased the time to first severe exacerbation compared to placebo, with an overall reduction of 21% (*P* = 0.03).^59^

#### *Bronchial thermoplasty*

Bronchial thermoplasty lowers exacerbations in patients with moderate‐to‐severe asthma not well controlled on ICS/LABA. However, its use in patients with severe disease and poor lung function, or high exacerbation risk (>4 steroid bursts or >1 exacerbation in 12 months) remains unclear.^60^, ^61^ Chupp *et al*. reported 3‐year follow‐up results from subjects treated with bronchial thermoplasty in the PAS2 study (post‐marketing trial, *n* = 190) and AIR2 (randomized control trial, *n* = 190).^62^ There was no change in percentage of patients taking maintenance OCS at baseline compared to 3 years later (4.2% vs 3.7%, respectively) but slight reduction in mean OCS dose (11.9 vs 7.3 mg prednisolone equivalent, respectively). In contrast, the PAS2 cohort which may better reflect ‘real‐world’ patients showed fewer patients needing maintenance OCS at 3 years (10% vs 19% baseline) but the median dose of OCS was increased from 9.1 to 14.6 mg prednisolone equivalent.

## BARRIERS TO AND SOLUTIONS FOR CHANGE

**Figure 2:**
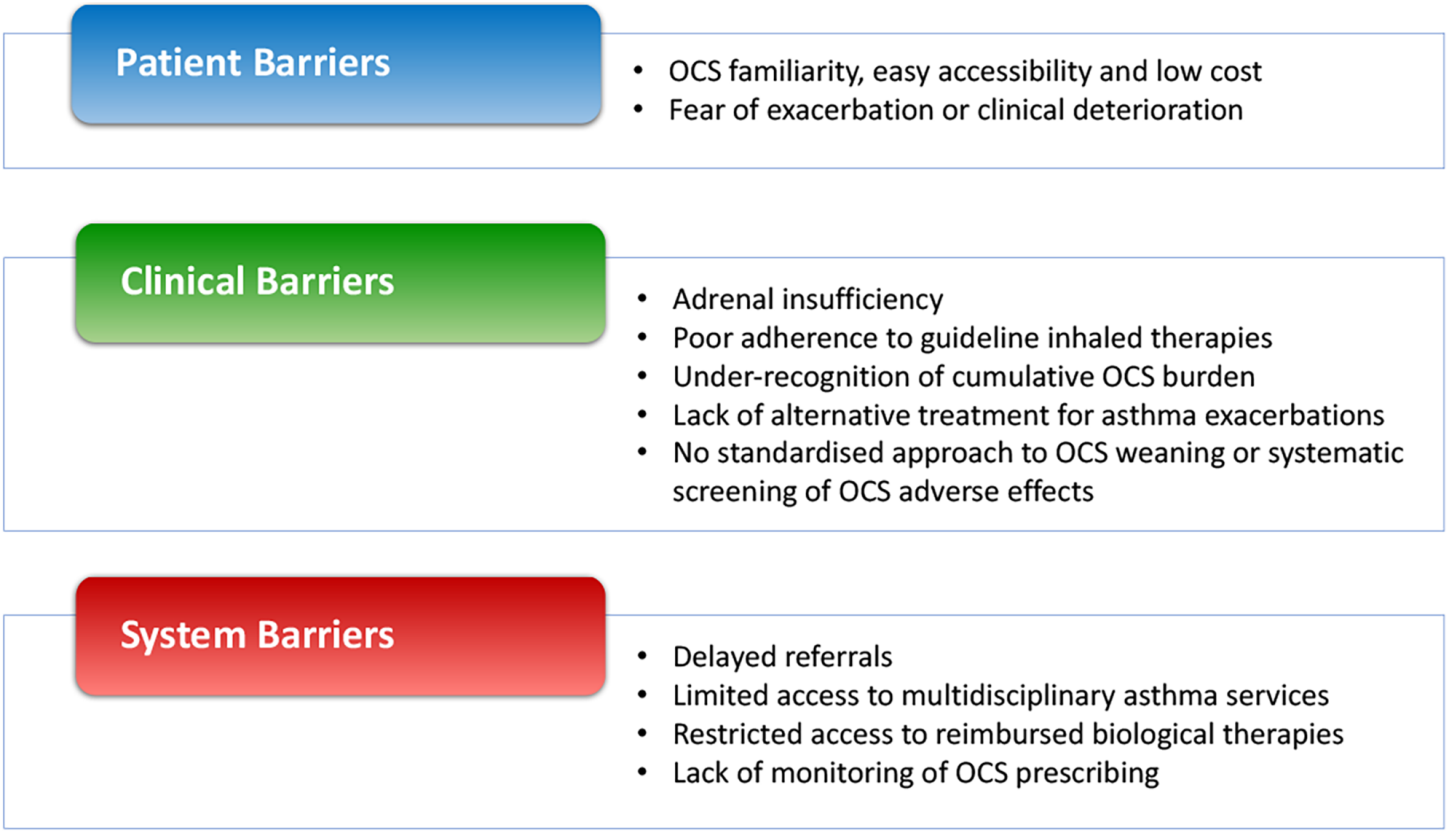
Potential barriers to reduce OCS exposure in severe asthma. OCS, oral corticosteroid.

A systematic approach addressing patient, prescriber and healthcare system barriers is required to reduce unnecessary OCS use and minimize unwanted side effects (Fig. 2). A substantial challenge, however, lies in the diverse group of clinicians who administer OCS, especially for acute use. Education campaigns highlighting OCS benefit and risk trade‐offs, and increased awareness of steroid‐sparing strategies are essential to change entrenched prescribing patterns.

### Patients' perspective

Patients typically overestimate asthma control, which may lead to excessive SABA use and delayed presentation to primary or tertiary care, increasing the risk of exacerbation.^63^ In a US survey, the majority of patients with asthma considered they were ‘well managed’ if they only required two urgent visits to their physician or hospital, or experienced <4 exacerbations per year.^64^ These patients, however, are at risk of cumulative AE associated with frequent OCS bursts.

Therefore, it is imperative that patients are educated about optimal asthma control and counselled about appropriate OCS use and its risk–benefit profile, whether taken as short bursts or as maintenance therapy.

### Clinical considerations

Several clinical factors hampering steroid reduction are discussed below.

#### *AI with maintenance OCS use*

Fear of adrenal insufficiency (AI) by clinicians may hinder steroid reduction in patients with severe asthma. A recent meta‐analysis showed AI can occur with any administration route, duration, dosing or underlying disease.^65^ AI was present in 43.7% of asthma patients treated with OCS versus 6.8% in those treated with ICS. The risk is greater when the dose exceeds 5 mg/day prednisolone (or equivalent) for >4 weeks.^65^, ^66^, ^67^ Random morning cortisol level <80 nmol/L is indicative of AI. A short Synacthen test is required for definitive diagnosis for those with an intermediate cortisol level.^67^, ^68^ Recovery from AI with prolonged OCS exposure is unpredictable, but usually takes several months, necessitating a slow OCS‐weaning regimen, even when steroid therapy is no longer necessary for asthma. Thereafter, patients with symptomatic OCS‐induced AI could be treated with glucocorticoid replacement therapy such as hydrocortisone.^67^, ^68^

#### *Suboptimal guideline adherence*

Overall, maintenance ICS for persistent asthma is under‐prescribed^39^ increasing the risk of exacerbations and the need for OCS bursts. GINA recommends daily prednisolone for up to 7 days in patients failing to respond to inhaler therapy escalation over 3 days; for those who rapidly deteriorate; or for people with a history of brittle exacerbations.^69^ However, action plans detailing a stepwise increase in asthma management are under‐utilized.^70^ Primary care providers are more likely to adhere to guideline recommendations if electronic reminders are embedded within practice software.^71^ Ongoing education through specialists and professional organizations is also important.

#### *Lack of effective alternatives for asthma exacerbations*

To date, OCS remains the best studied treatment for treating acute asthma exacerbations,^2^ with little evidence to suggest there are effective, alternative therapies other than a trial of ICS escalation.^6^ However, the recommended dose and duration vary between guidelines.^6^, ^31^

#### *Under‐recognition of cumulative burden*

Education campaigns are required to raise awareness of complications due to intermittent OCS use.^72^ Each exacerbation should prompt a thorough patient review to optimize asthma treatment plans. Patients requiring >2 courses of OCS/year should be referred for specialist assessment. Electronic alerts in prescribing systems could flag patients receiving multiple OCS courses and engaging allied health professionals, such as nurses and pharmacists, could help identify and educate patients at risk of OCS complications.

#### *No standardized approach for steroid weaning*

Currently, there are no standardized guidelines on how to safely taper OCS in patients with severe asthma.

Biological studies demonstrate effective steroid reduction using a pre‐set weaning protocol.^19^, ^20^ These patients were objectively assessed using Asthma Control Questionnaire (ACQ) and symptom diary monitoring for early symptoms of exacerbation. If patients' symptoms were stable, the prednisolone dose was reduced by 5–10 mg every 4 weeks until patients reached 10 mg/day, then the dose was further reduced to 7.5, 5, 2.5 and 1.25 mg/day on a monthly basis. In the event of clinical instability, the dose was returned to the level before deterioration. Notably, 23–55% of patients on placebo, who were previously deemed ‘steroid‐dependent’, were able to reduce their steroid dose by 50–75%, and approximately 10% were able to completely withdraw OCS use. These results suggest a lower steroid load can be achieved by simply adopting a more structured OCS‐weaning approach.

### System barriers and solutions

Widespread changes in OCS prescribing practice also require a change in healthcare systems and infrastructure.

#### *Delayed referrals*

A high proportion of patients with severe asthma do not receive specialist care due to under‐recognition of severe asthma, a lack of guideline awareness, delays in referral or other factors.^39^, ^64^ A cross‐sectional Australian survey showed only 10% of patients consulted a specialist for their condition despite a substantial asthma burden.^73^ To best triage patients with severe asthma, it is important for medical training programmes and materials to include specific referral criteria and guidance on critical information to include in a referral letter.^74^

#### *Limited access to multidimensional severe asthma services*

An estimated one in eight severe asthmatic patients already under the care of a specialist could further benefit from referral to an asthma service for MDA review.^75^ Severe asthma services are often limited by funding or resource restrictions including inadequate clinical or allied health staffing,^76^ and there is scope to improve collaboration and integration of severe asthma services with primary care.^74^

#### *Restricted access to biological therapies*

Access to biologicals is determined by local prescribing restrictions. In many countries, reimbursement criteria include pre‐specified OCS use over the preceding year,^18^ which may indirectly expose patients to excess steroid exposure. Additionally, the application process is often cumbersome, which may deter clinicians from prescribing biologics.^76^ There are, however, examples where clinicians have successfully lobbied for change in prescribing practice for better patient‐centred outcomes based on scientific evidence and rational clinical justification. For instance, in Australia, the initial mandatory requirement of being under the care of the same physician for 12 months was recently changed to either 6 months duration or assessment at a multidisciplinary clinic (http://www.pbs.gov.au/medicine/item/10980x-10996R-11003D-11014Q).

### Future directions to minimize OCS exposure

Additionally, further research and innovative strategies are required to prevent inappropriate OCS exposure in patients with severe asthma.

#### *Oral steroid stewardship*

**Figure 3:**
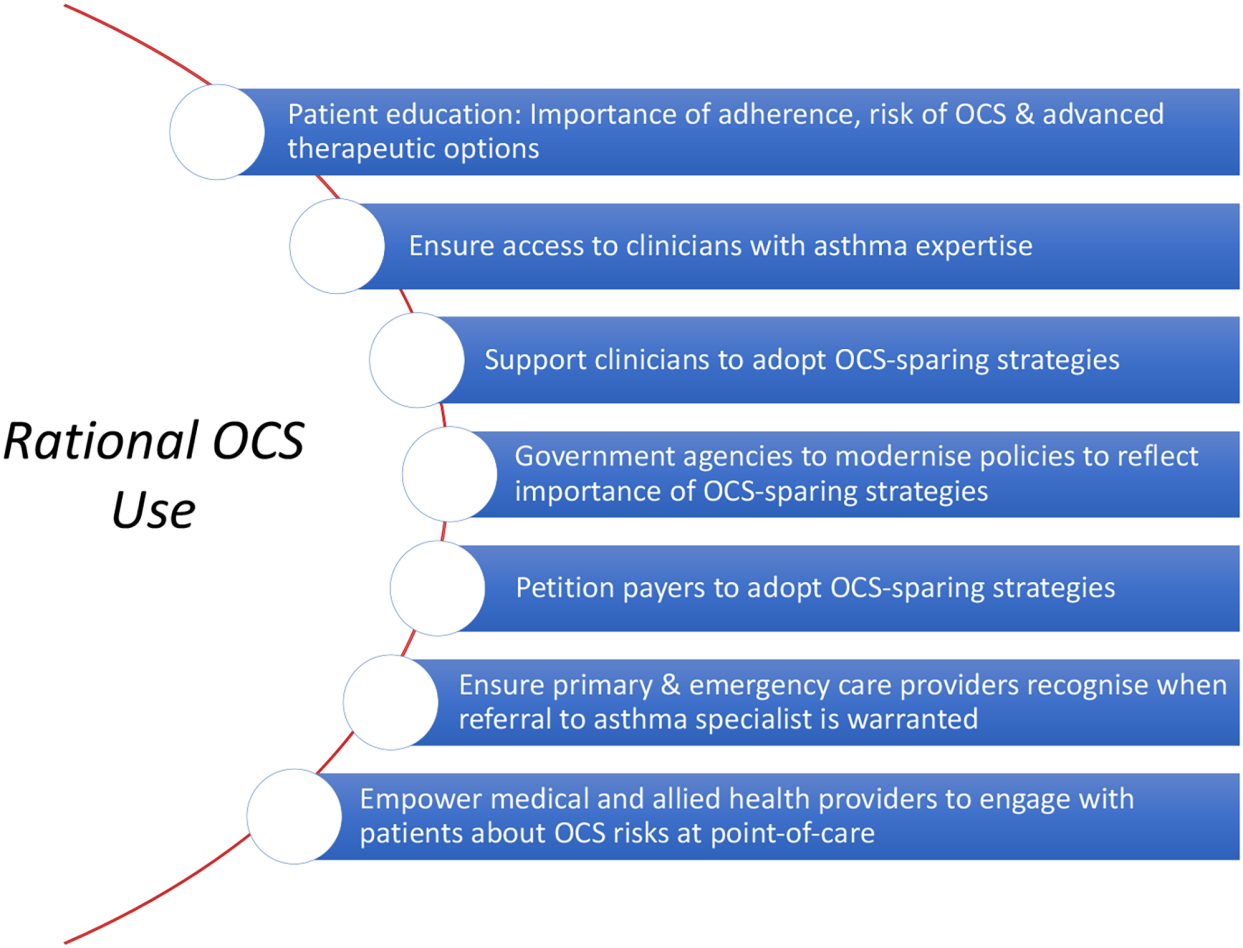
Key principles of OCS stewardship for asthma (Adapted from the Asthma and Allergy Foundation of America^77^). OCS, oral corticosteroid.

International respiratory experts are calling for ‘oral steroid stewardship’, which involves a structured approach for preventing and managing OCS exposure across multiple levels of the healthcare system.^76^ The Asthma and Allergy Foundation of America recently partnered with patient advocates, medical profession societies and industry partners to provide a stewardship statement to curb excess OCS exposure.^77^ A summary of these guiding principles is shown in Figure 3. The purpose of OCS stewardship is not to eliminate OCS use in asthma *per se*, where it still plays an important role, but to minimize inappropriate OCS use.

#### *Patient charter*

Key principles from the stewardship statement can be reiterated in a ‘patient charter’ aimed at improving patient care and outcomes in severe asthma. Core management goals include timely and equitable access to severe asthma services, individualized management specific to the asthma phenotype and reduced AE associated with OCS. These goals are achievable if there is universal adoption of the principles by patients, healthcare professionals and policymakers, supported by appropriate resource allocation.^25^

#### *Use of biomarkers prior to initiating maintenance steroids*

Before prescribing maintenance OCS, it is important to consider the likelihood of a clinical response, goals of therapy and risk of OCS‐related AE.

Typically, OCS treatment is commenced in patients with poor symptom control, irrespective of whether the underlying pathophysiology is steroid responsive. However, initiation and titration of long‐term OCS should occur on the basis of objective measures of steroid‐responsive airway inflammation (i.e. sputum or peripheral eosinophilia).^78^, ^79^ OCS effectively target type 2 eosinophilic inflammation, which is present in approximately 50% of patients with asthma. However, caution should be undertaken before introducing or escalating OCS in patients with non‐eosinophilic inflammation where OCS effect is less apparent.^79^, ^80^ These patients can be challenging to effectively treat if established on long‐term OCS. Given the favourable AE profile, biological therapies in well‐selected patients may be preferential to maintenance OCS for symptom control or preventing exacerbations.

#### *Biomarkers to tailor maintenance OCS*

Sputum eosinophil count was shown to be superior to routine clinical assessment alone in reducing asthma exacerbations, without the need for escalating asthma treatment.^81^, ^82^ Furthermore, OCS dose adjustment using peripheral eosinophil count in patients with severe treatment‐refractory asthma can provide superior symptom control, reduce exacerbations and lower maintenance OCS use.^83^

Maintenance OCS dose requirements over 6 months were compared using an internet‐based strategy and a conventional clinical assessment.^84^ Patients randomized to the first group received weekly instructions and nurse support for stepwise adjustments in OCS dose according to a pre‐determined algorithm using ACQ and mean fractional exhaled nitric oxide (FeNO) levels. In the internet‐based group, the prednisolone dose decreased from 10 to 5 mg/daily and approximately 20% of patients were completely weaned off prednisone. In contrast, the mean daily dose of prednisolone was increased by 1.6 mg in the conventional group. A randomized study is underway to compare composite biomarkers (i.e. blood eosinophils, periostin or FeNO) versus a standard symptom‐based strategy for adjusting steroid therapy.^85^

#### *Characterization of asthma exacerbations*

Guidelines recommend OCS for acute exacerbations but there is little guidance with regards to objective markers for exacerbations suitable for OCS. Exacerbations associated with elevated type 2 eosinophilic inflammation, measured by sputum or peripheral eosinophilia and/or FeNO, are most amenable to OCS therapy.^86^ The role of non‐type 2 inflammation during an exacerbation is less well understood, but may contribute to corticosteroid‐refractory response.^86^ Therefore, OCS as a one‐size‐fits‐all approach for exacerbations may be of limited value, but this requires further research. A better ability to phenotype asthma exacerbations, and an improved understanding of key mechanistic pathways and exploration of alternative treatment strategies, is crucial in ensuring appropriate use of short‐term OCS bursts.

## CONCLUSION

OCS for the treatment of asthma have been prescribed for more than 60 years and have conferred considerable benefit; however, these agents are associated with costly AE and complications. Given the scientific advances in treatments and biomarkers, as well as greater availability of specialized severe asthma clinics, it is prudent to judiciously re‐evaluate routine OCS use.

Further research and consensus are necessary for guidance on rational OCS prescribing, including down‐titration in the biological treatment era. Moreover, education and public health campaigns, which leverage government resources, are required to ensure that appropriate patients have access to specialized asthma services for treatment optimization and OCS‐sparing interventions.

In conclusion, it is incumbent on the medical profession to drive transformation in asthma care to ensure that OCS use is minimized where this is practical and safe. Every patient with severe asthma should receive individualized, safe and effective treatment to meet the goals of personalized medicine.

## Disclosure statement

L.P.C. has received speaker fees from AstraZeneca (AZ), GlaxoSmithKline (GSK), Boehringer Ingelheim (BI) and Menarini and served on selected advisory board meetings of GSK and AZ, unrelated to the current manuscript. J.W.U. has served on Advisory Boards for GSK, AZ, Novartis and BI, and has received speaker fees from GSK, AZ, Novartis and BI. His department has received unrestricted research and teaching grants from GSK and AZ, unrelated to the submitted work. P.G.B. has served on Advisory Boards for GSK, AZ, Novartis, BI and Sanofi. He has lectured at meetings arranged by GSK, AZ and BI, and his department has received unrestricted research and teaching grants from GSK and Novartis. M.H. reported receiving grants‐in‐aid, speaker fees and fees for serving on the advisory boards of GSK, AZ Novartis and Seqirus, unrelated to the current manuscript, all paid to his institutional employer Alfred Health. Medical writing support was provided by Celia Green, Bioscript Pty Ltd, which was funded by AZ Pty Ltd. However, AZ did not have any input into the final content of this publication.

## Author contributions

Conceptualization: L.P.C., J.W.U., P.G.B., M.H. Formal analysis: L.P.C., J.W.U., P.G.B., M.H. Funding acquisition: P.G.B. Methodology: L.P.C., J.W.U., P.G.B., M.H. Project administration: L.P.C., J.W.U., P.G.B., M.H. Supervision: L.P.C., J.W.U., P.G.B., M.H. Validation: L.P.C., J.W.U., P.G.B., M.H. Writing—original draft: L.P.C., J.W.U., P.G.B., M.H. Writing—review and editing: L.P.C., J.W.U., P.G.B., M.H.

**Abbreviations**
ACQ: Asthma Control Questionnaire
AE: adverse effect
AI: adrenal insufficiency
FeNO: fractional exhaled nitric oxide
GINA: Global Initiative for Asthma
HRQoL: health‐related quality of life
ICS: inhaled corticosteroid
IRR: incident rate ratio
LABA: long‐acting beta_2_‐agonist
MDA: multidimensional assessment
OCS: oral corticosteroid
OR: odds ratio
Q2W: every 2 weeks
Q4W: every 4 weeks
Q8W: every 8 weeks
SABA: short‐acting β_2_‐agonist
SC: subcutaneous
SCS: systemic corticosteroid

## Supporting information


**Table S1.** Prevalence of OCS use in asthma patients in Asia‐Pacific Region.
