# Supplementary material for: Rational oral corticosteroid use in adult severe asthma: A narrative review
Source: Respirology. 2019 Nov 12;25(2):161–72. doi: 10.1111/resp.13730 (PMC7027745; doi:10.1111/resp.13730)
Supplement: Supplementary file 1 — Table S1. Prevalence of OCS use in asthma patients in Asia‐Pacific Region. [file RESP-25-161-s001.docx]

**SUPPLEMENTARY INFORMATION**

**Rational oral corticosteroid use in adult severe asthma: A narrative review**

Chung L.P.^1^, Upham JW^2^, Bardin P.G.^3^, Hew M^4^.

1. Respiratory Physician, Fiona Stanley Hospital, Perth, WA, Australia
2. Respiratory Physician, Princess Alexandra Hospital, and The University of Queensland, Brisbane, QLD, Australia
3. Respiratory Physician, Monash Medical Centre and Monash University, Melbourne, VIC, , Australia
4. Respiratory Physician, Allergy, Asthma & Clinical Immunology, Alfred Hospital, VIC, , Australia

**Table S1: Prevalence of OCS use in asthma patients in Asia Pacific Region**

| **Country** | **Prevalence of OCS use in the previous 12 months** |
| --- | --- |
| Australia | 35% |
| Singapore | 26% |
| Thailand | 40% |
| South Korea | 41% |
| Taiwan | 43% |
| Malaysia | 51% |
| China | 72% |
| India | 89% |

This is cross-sectional data summarized from 3630 asthma patients from the Asia Pacific Insights and Management Survey.^23^ The patients were characterized based on level of asthma control as defined by the GINA guidelines.
